# The role of thrombospondin-1 in dermatological conditions

**DOI:** 10.3389/fmed.2026.1724955

**Published:** 2026-03-18

**Authors:** Li-shanjie Chen, Bo-wen Zheng, Chuan-ying Zhao, Cong-cong He, Xing-Hua Gao

**Affiliations:** 1 Department of Dermatology, The First Hospital of China Medical University, Shenyang, China; 2 Key Laboratory of Immunodermatology, Ministry of Education, and National Health Commission, National Joint Engineering Research Center for Theranostics of Immunological Skin Diseases, Shenyang, China

**Keywords:** angiogenesis, fibrosis, microenvironment, skin, thrombospondin-1

## Abstract

Thrombospondin-1 (THBS1) is a multifunctional extracellular matrix protein that interacts with various cell surface receptors, including cluster of differentiation 36 (CD36), CD47, and integrins, as well as secreted proteins such as angiogenic factors and transforming growth factor (TGF)-*β*. THBS1 drives the development of various skin diseases at the molecular level by modulating core pathological processes, such as angiogenesis, fibrosis, inflammation, and tumor progression, thereby serving as a pivotal nexus between fundamental research and clinical dermatology. This review systematically explores the molecular mechanisms of THBS1 signaling in the skin, highlighting its implications in wound healing, tissue regeneration, inflammatory responses, fibrosis, and skin tumor development. By examining these aspects, we aim to elucidate the multifaceted roles of THBS1 in clinically refractory skin diseases and its translational potential as a therapeutic target.

## 1 Introduction

Thrombospondin-1 (THBS1), also referred to as TSP-1, is a constituent of the thrombospondin family. THBS1 was initially identified in platelets in 1990 and recognized as an endogenous inhibitor of angiogenesis (1–3). The thrombospondin family comprises five protein-encoding genes, designated as THBS1-5. These proteins are categorized into groups A and B. Group A THBS proteins (THBS1 and THBS2) exist as homologous trimers, consisting of three identical subunits linked by interchain disulfide bonds and containing transcriptional regulatory sequences (TSRs). Group B THBS proteins (THBS3, THBS4, and THBS5) are assembled in pentamer form and lack TSRs (4). Among them, THBS1 is the only member of the THBS family with a clear ability to activate potential transforming growth factor (TGF)-*β* function through its TSR, specifically via GGWSHW and RFK sequences (5).

**Figure 1:**
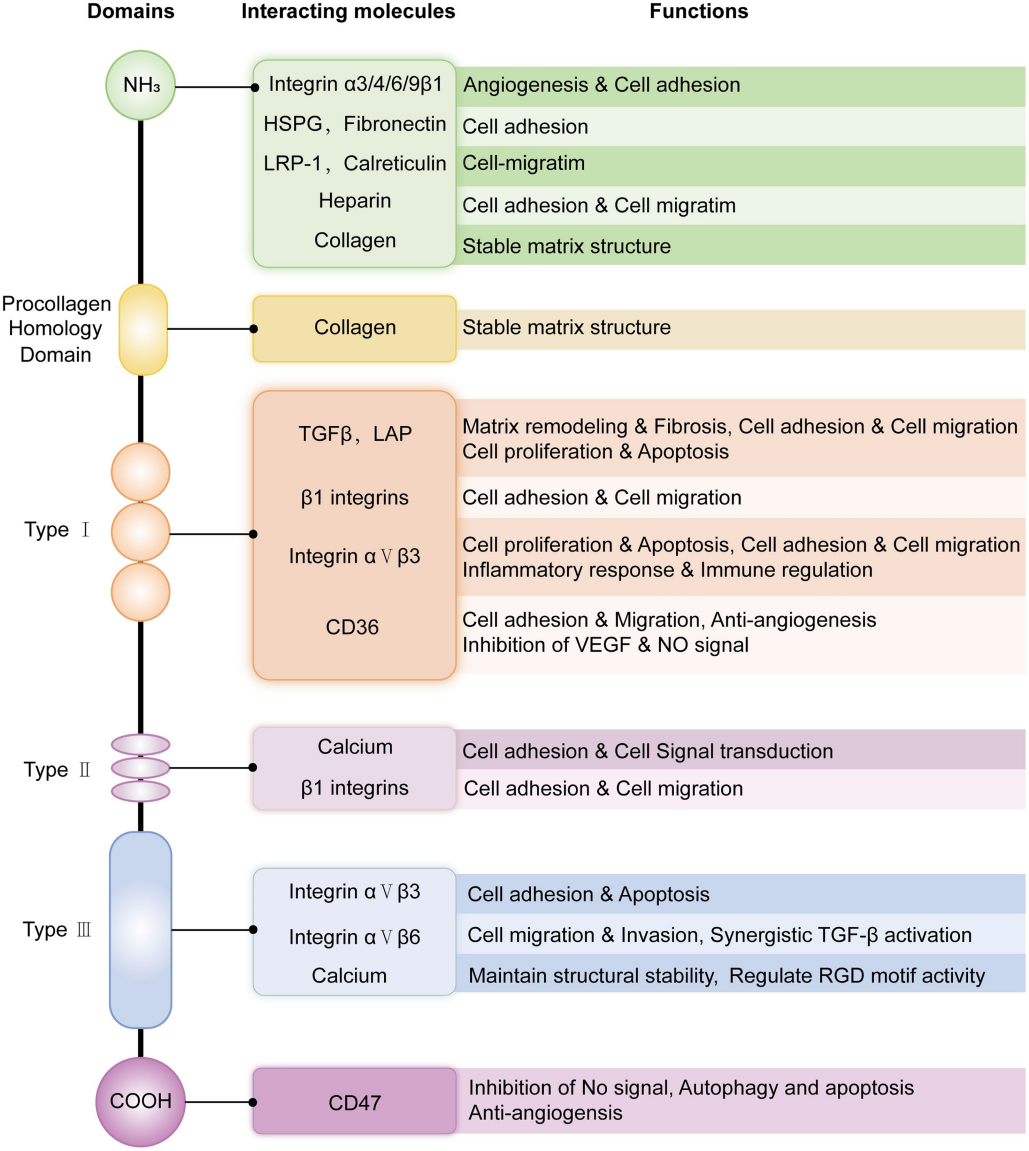
Schematic structure and main interacting molecules of THBS1 and related functions. The heparin-binding site of the N-terminal domain mediates the binding of heparan sulfate proteoglycans (HSPGs), promotes cell adhesion, and participates in cell-matrix interactions and growth factor recruitment. The procollagen domain binds collagen through hydrophobic interactions, stabilizes the matrix structure, and regulates extracellular matrix assembly and cell migration. The type 1 repeat domain (TSR) can bind to CD36, fibrinogen, and latent-associated peptide (LAP), activate TGF-β, and regulate anti-angiogenesis, inflammatory responses, and matrix remodeling. The type 2 repeat domain (EGF-like repeat) can bind integrins (such as α3β1 and α6β1) and promote cell adhesion and migration. Type 3 repeat domains, homologous to calcium-binding sites, also known as calcium-binding repeats, bind to integrins such as αvβ3 via RGD (arginine-glycine-aspartic acid) motifs to promote cell adhesion and apoptosis. The C-terminal domain can bind to CD47, affecting cell migration and apoptosis.

THBS1 is a six-domain glycoprotein: an N-terminal signal peptide, a procollagen homology domain, three repeat domains (TSR, EGF-like, and TSP Ca-binding), and a C-terminal domain (6). THBS1 is produced by various normal cells (e.g., fibroblasts, endothelial cells, and macrophages) and some tumor cells (e.g., squamous cell carcinoma, melanoma, and lymphoma) (7). As a typical secreted protein, THBS1 interacts with a variety of molecules through its six domains, fulfilling its core functions in angiogenesis, cell adhesion (8, 9), immune regulation, and extracellular matrix (ECM) remodeling (10) (Figure 1). The specific binding of different molecules to the THBS1 domain provides a key site for the development of targeted therapies. In addition, THBS1 can enter the nucleus for transcriptional regulation after being cut and modified by matrix metalloproteinases and endocytosis under specific pathologies (such as hypoxia and inflammation) (11). However, the specific structural and functional differences between secretion and nuclear entry are still under investigation and remain unclear.

Currently, an increasing number of studies are focusing on the role of THBS1 in the skin. In conditions of skin fibrosis, under the influence of interleukin 6 (IL-6), fibroblasts overmultiply and secrete THBS1, which promotes collagen synthesis by activating the TGF-*β* signaling pathway, leading to collagen deposition (12). In chronic skin inflammation, pro-inflammatory cytokines tumor necrosis factor (TNF)-*α* and IL-1β stimulate the expression of THBS1 in fibroblasts and immune cells to regulate macrophage polarization, enhance cell adhesion, and regulate immune response (13, 14). In injured skin, platelets are activated and release THBS1, participating in the coagulation and repair processes (15). THBS1 also inhibits endothelial cell migration by binding to CD36 and CD47, limiting angiogenesis. In skin tumors, THBS1 inhibits angiogenesis by binding to CD36 to limit tumor growth, but its interaction with CD47 and integrins can inhibit macrophage activation, prevent tumor cells from being phagocytosed, and promote the metastasis of cutaneous T-cell lymphoma and melanoma cells (16, 17). In regard to hair growth, the secretion of THBS1 by dermal papilla cells and fibroblasts around hair follicles significantly increases, promoting the proliferation and differentiation of secondary hair follicle cells by activating the MAPK signaling pathway (18). The role of THBS1 in skin diseases involves a complex regulatory network of multiple cells and multiple pathways, and its secreting cells and production conditions (hypoxia and inflammatory factors) are closely related to the skin pathological process (Table 1). However, existing reports are often scattered “phenomenological descriptions,” lacking systematic integration and mechanistic elucidation of the complex and contradictory functions of THBS1 in the skin. This review summarizes the latest research on the roles and molecular functions of THBS1 in various skin diseases, aiming to clarify the complex, contradictory mechanisms underlying THBS1 in the skin, develop targeted intervention strategies, and facilitate its translation from basic discoveries to clinical applications.

**Table 1:** Signal pathway, receptors, and downstream signals of THBS1 and related dermatologic diseases.

Signal pathway	Receptor	Downstream signal	Function	Relevant skin diseases	Reference
PI3K/AKT	CD36	NO	Inhibition of angiogenesis and destruction of the vascular environment required for tumor growth and metastasis	Melanoma	(127)
	CD47	Vascular endothelial growth factor receptor 2 (VEGFR2)	Inhibition of VEGFR2 downstream target SRC phosphorylation in T cells, thus antagonizing T-cell migration and activation	Melanoma	(17)
	CD47	Nitric oxide (NO)	Blocking endothelial cell adhesion, inhibiting skin blood flow and angiogenesis; promoting tumor cell metastasis and development	Skin flap transplantation, melanoma	(25, 29)
	CD47	AKT	Promotion of tumor cell proliferation and survival, induction of cell migration and growth *in vivo*	Cutaneous T-cell lymphoma	(16)
	Platelet-derived growth factor receptor (PDGFR)	AKT	Activation of fibroblast proliferation, migration, and ECM production	Systemic sclerosis, scarring	(99, 102)
JAK-STAT	IL-6	STAT3	Promotion of fibroblast proliferation and collagen secretion	Scarring	(12)
	IL-10	STAT3	Induction of malignant transformation and promotion of the proliferation, escape, and metastasis of tumor cells	Photocarcinogenesis	(81)
TGF-β/SMAD	TGF-β	Smad2/3	Restoring cell vitality, delaying aging and death; Increasing collagen expression; Inhibition of neutrophil and macrophage migration and Th17 differentiation	Photoaging; Skin fibrotic diseases; Cutaneous inflammation	(64, 65, 128)
MAPK signaling pathways	FGFR	ERK	Promotion of hair follicle organ development	Alopecia	(18)
	CD47	ERK1/2	Promotion of proliferation, migration, and survival of tumor cells	Cutaneous T-cell lymphoma	(16)
	α9β1 α3β1 α4β1 α6β1	ERK1/2	Promotion of angiogenesis, tumor cell adhesion, and migration	Cutaneous inflammation; cutaneous tumor	(129, 130)
	PDGFR	ERK1/2	Activation of fibroblast proliferation, migration, and ECM production	Systemic sclerosis; scarring	(102)

## 2 Mechanism of THBS1 signal regulation

THBS1 interacts with a variety of receptors and molecules to integrate extracellular signals and intracellular responses, involving in an array of skin physiological and pathological processes.

### 2.1 Receptors

#### 2.1.1 CD36

CD36 plays a dual role, promoting and suppressing tumor growth. The interaction between the TSR domain of THBS1 (which contains the cysteine-serine-valine-threonine-cysteine-glycine [CSVTCG] sequence) and the CLESH domain of CD36 primarily leads to tumor suppression and inhibition of angiogenesis (2). However, in acute myeloid leukemia (AML) and prostate cancer, THBS1-CD36 appears to promote tumor metastasis, likely due to its role in creating a hypoxic environment by inhibiting angiogenesis (19). Meanwhile, the study found that in advanced tumors (such as colon cancer and liver cancer), THBS1-CD36 activates the TGF-*β*/Smad pathway, upregulates epithelial-mesenchymal transition (EMT) transcription factors such as Snail and Twist, and remodels the extracellular matrix (ECM) through integrin synergy, enhancing tumor cell invasion and promoting the formation of metastatic foci (20, 21). Therefore, the interaction between THBS1 and CD36 is highly dependent on the tumor type and microenvironment, and plays a dual regulatory role in tumorigenesis and metastasis. It inhibits tumor progression through anti-angiogenesis and pro-apoptosis in the early stage, but may promote metastasis and immune escape in the late stage.

#### 2.1.2 . CD47

Following O-fucosylation and C-mannosylation modifications, THBS1 undergoes conformational changes that enhance its binding affinity to CD47 via the C-terminal RFYVVMWK sequence (4N1K) within its CTD domain (22). In human umbilical vein and microvascular endothelial cells, VEGFR2-Y1175 phosphorylation can recruit phospholipase Cγ (PLCγ), and then activate downstream Erk1/2 signaling and PI3K/AKT (Phosphoinositide 3-kinase/Protein Kinase B) signaling pathways, promoting the proliferation, differentiation, migration, and formation of tubular structures of endothelial cells (23, 24). After THBS1 binds to CD47, it inhibits the phosphorylation of VEGFR2-Y1175, thereby blocking the signaling of VEGF induced by angiogenic factors and exerting a powerful anti-angiogenic effect (25, 26). Furthermore, THBS1-CD47 promoted cellular senescence (27). The concentration of THBS1 in tissues increases with age, rising in senescent cells as the ECM becomes less elastic and more rigid. Proliferative decline and cell cycle arrest are hallmarks of cellular aging. Compared to age-consistent wild-type mice, there is a significant reduction in the abundance of proliferating cell nuclear antigen (PCNA) in THBS1 knockout mice (28). Mechanically, blocking antibodies against CD47 diminish the production of superoxide (a form of reactive oxygen species) induced by THBS1 in human pulmonary artery endothelial cells, thereby delaying senescence. Additionally, THBS1-CD47 interaction restricts nitric oxide signaling, reduces skin blood flow, and inhibits tissue survival. Blocking this interaction with antibodies or antisense morpholino oligonucleotides significantly improves skin graft survival (29, 30).

CD47, an immunoregulatory molecule, is highly expressed in several types of tumor cells (e.g., colorectal cancer and ovarian cancer). In JURKAT T cells, THBS1 binding to CD47 inhibits VEGFR2 signaling, resulting in decreased phosphorylation of SRC, a downstream target of VEGFR2, thereby antagonizing VEGF-induced T-cell migration and activation (31). Furthermore, the THBS1-CD47 interaction inhibits macrophage activation and prevents tumor cells from being phagocytosed; this process inhibits nitric oxide signaling and accelerates the formation and activation of osteoclasts. Osteoclasts secrete matrix metalloproteinases (MMPs) and proteolytic enzymes to degrade the ECM and promote the metastasis and progression of bone tumor cells (32, 33). Compared to wild-type mice, sections of B16 melanoma tumors grown in a CD47-deficient microenvironment exhibited doubled expression of granzyme B mRNA, a significant 60% increase in CD8 + T-cell infiltration, and a 45% reduction in tumor wet weight, effectively inducing tumor cell death (34). It has been speculated that metastatic melanoma cell subpopulations may rely on CD47 expression for immune evasion, making the strategy of blocking the THBS1/CD47 axis an innovative target for tumor therapy (35).

#### 2.1.3 Integrin

Integrins constitute a family of heterodimeric membrane proteins, characterized by non-covalent complexes formed from a single *α* chain and a *β* chain (36). Multiple domains of THBS1 bind to integrins and play a role in angiogenesis, inflammatory repair and tumor immunity.

Integrins α9β1, α3β1, and α4β1 bind to the NH2 domain of THBS1, promoting angiogenesis via the Erk1/2 and paxillin pathways (37). In inflammatory colitis, THBS1 in endothelial cells binds to αvβ3 integrin, leading to nitric oxide production, vasodilation, and intussusception angiogenesis (38). In rat vascular smooth muscle cells, cyclic stretch boosts THBS1 expression and secretion, which binds to integrin αvβ1 and co-localizes with p-paxillin. This process regulates Yes-associated protein (YAP) nuclear shuttling via rap2, triggering vascular remodeling (39).

Integrins facilitate leukocyte rolling, adhesion, crawling, and transendothelial migration. THBS1 binds to integrins, aiding in inflammation and injury repair (40, 41). During early renal ischemia–reperfusion injury, extracellular vesicles with integrin β1 attract Fn1 + macrophages. These macrophages release THBS1, which binds to integrin β1, enhancing inflammatory factor expression, macrophage adhesion, migration, and amplifying inflammation in a concentration- and time-dependent manner (42). In corneal wounds, integrin αvβ6 binds to the RGD sequence of latent TGF-β1 potential LAP, causing LAP degradation and TGF-β1 activation, which promotes fibrotic wound healing and speeds up recovery of the mouse matrix and epithelium (43). In patients with glomerulosclerosis, levels of THBS1 and integrin β3 are elevated in tubular cells, and blocking the integrin-TGF-β1 interaction has an effective anti-fibrotic effect (44).

In tumor cells, integrin overexpression and signaling enhance adhesion, migration, invasion, immune evasion, and tumor growth (45, 46). In lung cancer, THBS1 overexpression in resistant strains boosts mmp2 and mmp9 expression via integrin signaling, promoting invasion and epithelial-mesenchymal transition (EMT) (47). THBS1, a target gene of YAP, plays a pivotal role in promoting melanoma cell invasion and may contribute to TGF-*β* activation, although its specific interaction with αvβ8 integrin remains to be fully elucidated (48). Notably, in melanoma, overexpression of αvβ8 integrin in regulatory T cells (Tregs) activates TGF-β signaling, which facilitates tumor immune evasion and may potentially interact with THBS1-mediated pathways (49). RGD-conjugated nanomaterials and gold nanoshell-RGD peptides, which target integrin-associated pathways, have demonstrated therapeutic potential for eradicating melanoma cells (50).

THBS1 exerts a canonical yet complex dual-edged effect on angiogenesis, tumor immunity, and tissue homeostasis by engaging receptors such as CD36, CD47, and various integrins. Its ultimate biological function is highly dependent on the specific cellular context, pathological stage, and the dynamically evolving tumor microenvironment (TME). During early tumorigenesis, THBS1 binding to CD36 activates caspase-3 and induces Fas/TRAIL-dependent endothelial apoptosis (2), while simultaneously recruiting the phosphatase SHP1 to inhibit VEGFR2 phosphorylation (51), thereby blocking the PI3K-AKT/eNOS signaling axis and exerting potent anti-angiogenic and tumor-suppressive functions (52, 53). In contrast, in advanced tumors or under hypoxic and inflammatory conditions, sustained vascular inhibition stabilizes HIF-1α and induces epithelial-mesenchymal transition (EMT) (54, 55). Meanwhile, post-translationally modified THBS1 binds with high affinity to CD47, delivering a “do not-eat-me” signal that suppresses macrophage phagocytosis and T-cell activation (56), promotes reactive oxygen species (ROS) generation, and inhibits NO signaling, thereby driving cellular senescence and immune escape—collectively fostering tumor progression (57). Furthermore, through distinct structural domains, THBS1 interacts with multiple integrins to activate downstream pathways such as FAK/Erk and Rap2/YAP, promoting endothelial cell migration, vascular remodeling, and extracellular matrix degradation, which contributes to pro-angiogenic and pro-invasive roles in wound healing, chronic inflammation, and certain tumor contexts (39). This high degree of context-dependency stems from the dynamic competition and integration of downstream signaling networks activated by its engagement with different receptors, posing both profound challenges and unique opportunities for developing context-guided, precise therapeutic strategies targeting THBS1 and its associated pathways.

### 2.2 Secretory proteins

#### 2.2.1 Angiogenic factors

**Figure 2:**
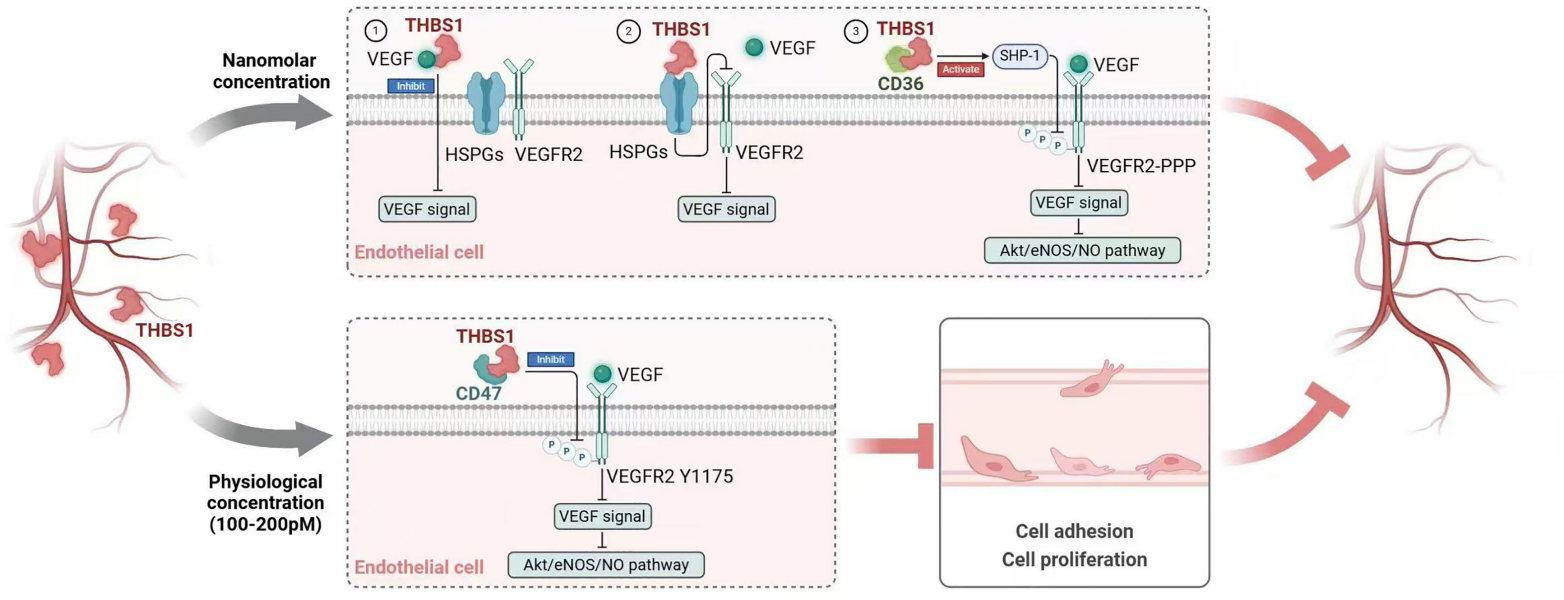
Mechanism of angiogenesis inhibition with different THBS1 concentrations. At nanomolar levels, THBS1 can bind to VEGF directly via its type 1 repeats and procollagen domain, sequestering VEGF and preventing it from promoting angiogenesis. It can also competitively bind to VEGF co-receptor cell surface heparan sulfate proteoglycans (HSPGs), disrupt the interaction between VEGF and VEGFR2, and inhibit VEGF signaling. Additionally, THBS1 interacts with CD36 to activate SHP-1, which is the phosphatase of VEGF2. This interaction subsequently inhibits VEGF-related signaling pathways, including the AKT/eNOS/NO pathway. In physiological plasma concentrations (100–200 pM), THBS1 competitively binds CD47 and inhibits the phosphorylation of VEGFR2-Y1175, thereby inhibiting NO production and exerting an anti-angiogenic effect.

Angiogenesis is crucial for embryogenesis and wound healing, driven by factors like VEGF, fibroblast growth factor (FGF), and platelet-derived growth factor (PDGF). VEGF and bFGF recruit endothelial cells, while PDGF stabilizes new vessels (58). THBS1, despite being an angiogenesis inhibitor, has complex, context-dependent effects that both promote and inhibit angiogenesis. The inhibitory effects of THBS1 on VEGF and anti-angiogenesis vary based on its concentration. At nanomolar concentrations, THBS1 can directly bind to VEGF or interact with receptors, including heparan sulfate proteoglycans and CD36, thereby inhibiting VEGF signaling (59, 60). At physiological plasma concentrations (100–200 p.m.), THBS1 exerts an anti-angiogenic effect by inhibiting the phosphorylation of VEGFR2 (Figure 2) (25, 61). However, the N-terminal heparin-binding domain of THBS1 was found to promote angiogenesis. In some tumor microenvironments (such as melanoma), THBS1 transcriptional activity is increased, with increased expression and secretion, promoting angiogenesis and metastasis of tumor cells. Mechanically, THBS1 can competitively bind to syndecan-4 on endothelial cell surfaces, activate protein kinase C through the PI3K/AKT signaling pathway, promote endothelial cell proliferation, migration, tubular structure formation, and MMPs expression, and then degrade the extracellular matrix and create favorable conditions for angiogenesis and tumor metastasis (62). Syndecan-4 is the primary mediator of THBS1’s role in facilitating angiogenesis. However, the specific mechanisms by which THBS1 promotes angiogenesis and tumor migration while bypassing vascular inhibition in the tumor environment are not well understood. Some studies suggest this involves a local balance of VEGF and TGF-*β* activated by THBS1.

#### 2.2.2 TGF-*β*

TGF-β plays a crucial role in various cellular processes, including cell proliferation, phenotypic conversion, migration, metabolism, and immune surveillance. It is instrumental in regulating embryonic development, maintaining tissue homeostasis, and facilitating injury repair (63). THBS1 contributes to extracellular matrix formation and modulates the activity and effects of the TGF-*β* signaling pathway. Typically, TGF-β is sequestered in the extracellular matrix through its association with LAP and becomes active and capable of receptor activation only upon release from its latent complex. THBS1 possesses an exposed peptide sequence lysine-arginine-phenylalanine-lysine (KRFK) that can interact with the conserved sequence leucine-serine-lysine-leucine (LSKL) in the LAP of all TGF-*β* isoforms. This interaction disrupts the binding of LAP to the sequestered TGF-β, thereby modulating the TGF-β signaling pathway (5). Following TGF-β activation of THBS1, fibroblast proliferation is stimulated, and collagen synthesis is enhanced through both SMAD-dependent and non-dependent signaling pathways (64). Concurrently, this process inhibits the synthesis of MMPs, reduces collagen degradation, promotes skin fibrosis, and mitigates photoaging (65).

## 3 THBS1 and ultraviolet light

**Figure 3:**
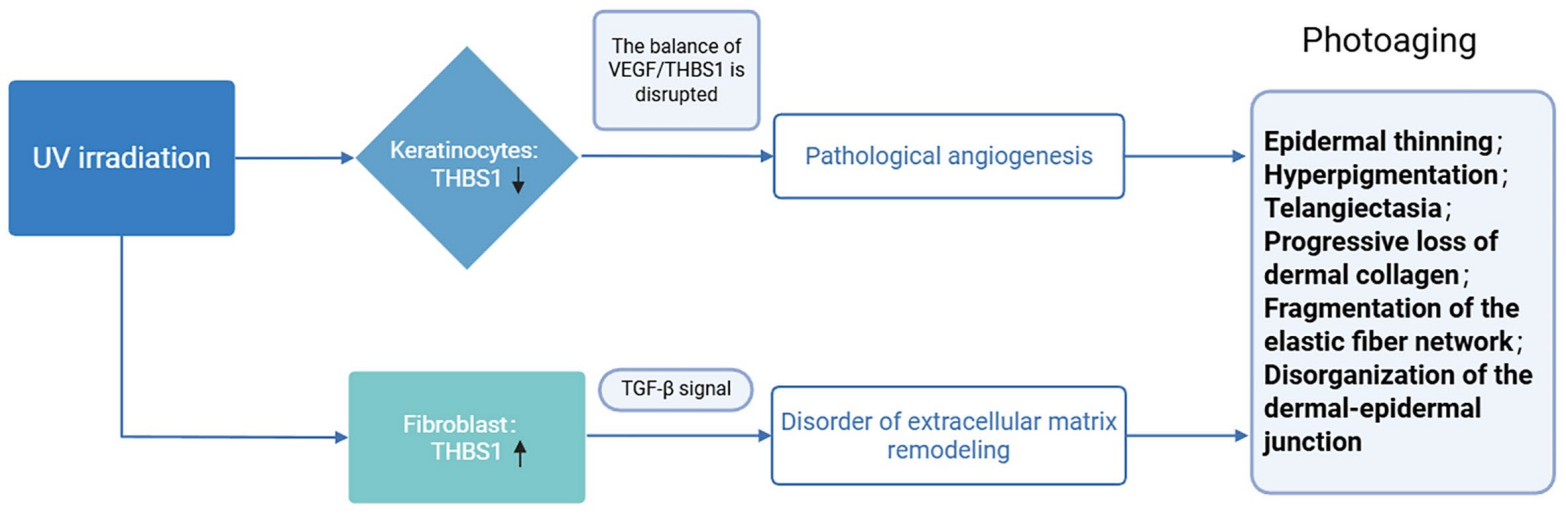
The function of THBS1 under ultraviolet light irradiation.

As a critical barrier organ, the skin is the primary target of solar ultraviolet radiation. Prolonged exposure to ultraviolet B (UVB) radiation can result in the degradation of matrix macromolecules, a reduction in skin elasticity, and the formation of wrinkles. These changes contribute to photoaging and elevate the risk of skin cancer. Angiogenesis is a significant factor in mediating ultraviolet (UV)-induced skin damage (66, 67). As an established angiogenesis inhibitor, THBS1 mitigates UVB-induced angiogenesis, inflammatory cell infiltration, and skin photodamage by modulating the equilibrium between pro-angiogenic and anti-angiogenic factors in the skin (68). This regulatory mechanism consequently diminishes the occurrence of precancerous lesions and malignant tumors. Notably, under UV irradiation, the expression, function, and ultimate biological effects of THBS1 exhibit high cell-type specificity as well as temporal- and dose-dependent dynamics. In keratinocytes, THBS1 predominantly promotes apoptosis, partly through its association with CD36 and related death receptor-associated pathways, thereby contributing to the elimination of potentially cancerous mutated cells (69). In fibroblasts, THBS1 interacts with integrins such as αvβ1 to transmit signals that inhibit migration and promote matrix degradation, exacerbating skin damage (70). In melanocytes, with prolonged UV exposure, the expression and function of THBS1 gradually shift from maintaining homeostasis toward promoting malignant transformation and tumor progression. Early and moderate activation of THBS1 signaling drives cell proliferation, migration, and orderly extracellular matrix synthesis, which supports tissue repair and regeneration. Conversely, sustained overactivation of THBS1 signaling leads to cellular senescence, chronic inflammation, and abnormal degradation or excessive deposition of the extracellular matrix, thereby aggravating photoaging (Figure 3).

### 3.1 Angiogenesis

UV radiation can instigate an angiogenic switch and modulate the balance between positive and negative regulators of skin angiogenesis (71). The modulation of THBS1 expression by UV radiation is contingent upon the specific cell type involved. In the epidermis, acute UV exposure activates the PI3K-AKT cell survival pathway via the epidermal growth factor (EGF) receptor, resulting in AKT phosphorylation and a reduction in THBS1 expression in keratinocytes. Concurrently, VEGF levels increase, disrupting the balance between VEGF and THBS1 expression and transforming the skin vascular environment into a pro-angiogenic state (72, 73). Research indicates that mice whose basal epidermis and outer root sheath follicular keratinocytes overexpress THBS1 have reduced skin damage, wrinkles, and angiogenesis under chronic UV exposure (74). In dermal fibroblasts of human skin, UV irradiation elevates THBS1 mRNA and protein expression through the PI3K-AKT–mTOR pathway, thereby inhibiting angiogenesis (75). Notably, THBS1 expression in mouse mesenchymal stem cells remains unchanged under ultraviolet irradiation (76).

### 3.2 Photoaging

The structural alterations observed in cutaneous photoaging manifest as multiple histopathological changes: attenuation of epidermal layers, disorganization of the dermo-epidermal junction, progressive loss of dermal collagen, fragmentation of elastic fiber networks, dyschromic manifestations, and vascular ectasia. Ultraviolet radiation can induce apoptosis in skin cells via intrinsic or extrinsic pathways, which encompass direct DNA damage, the production of reactive oxygen species (ROS), and the activation of cell-membrane death receptors (77). Prolonged exposure to UV radiation results in the activation of human elastase, degradation and loss of elastic fibers, denaturation and deformation of dermal elastin, and pronounced dermal angiogenesis. These processes collectively contribute to reduced skin elasticity and the formation of wrinkles, indicative of photoaging. THBS1 activates the TGF-*β*/Smad pathway by suppressing inflammation, proliferation, and angiogenesis. It alleviates the UV-induced downregulation of type I procollagen expression in human dermal fibroblasts, thereby restoring collagen formation and preventing UV-induced angiogenesis and skin photodamage. Research indicates that VEGF expression is upregulated in epidermal keratinocytes following acute UVB irradiation, whereas THBS1 expression is significantly downregulated, which increases the production of ROS, inhibits the proliferation of fibroblasts, and reduces the ability to synthesize collagen, thereby inducing photodamage (72, 73). In addition, under UVB irradiation, mice that specifically overexpress THBS1 exhibit significantly reduced skin vascularization, diminished endothelial cell proliferation, increased endothelial cell apoptosis, elevated p-Smad2/3 protein expression, and decreased skin photodamage and wrinkle formation (74). Based on these findings, Li et al. proposed a novel and promising approach for treating photoaging and promoting skin regeneration. This method uses skin-derived precursor-conditioned medium (SKP-cm), which mitigates photodamage by early activation of the TGF-*β*/Smad signaling pathway via THBS1 (65).

### 3.3 Photocarcinogenesis

The neovascularization process mediates tumor nutrient acquisition while creating migratory pathways for neoplastic dissemination to distant sites. THBS1 is an important autocrine factor that inhibits tumor angiogenesis by inhibiting endothelial cell proliferation and chemotaxis and depleting circulating endothelial progenitor cells (78, 79). In normal skin, THBS1 is highly expressed, particularly in epidermal keratinocytes. Overexpression of THBS1 in the epidermis of mice has been shown to inhibit angiogenesis and decrease the incidence of squamous cell carcinoma (80). Following UV irradiation, the expression of THBS1 in both mouse and human epidermis is markedly reduced. This reduction not only promotes angiogenesis but also induces the expression of IL-6, IL-10, and Janus kinase 1 (JAK1), subsequently activating the signal transducer and activator of transcription 3 (STAT3) signaling pathway, which is crucial for inhibiting tumor lesion proliferation and cell apoptosis. Additionally, caffeic acid (CA) has been shown to prevent THBS1 loss in the skin of UVB-irradiated mice, while apigenin can restore THBS1 expression via the mRNA-binding protein HuR, offering protection against UVB-induced photocarcinogenesis (80, 81).

## 4 THBS1 in skin tissue healing, regeneration, and transplantation

Skin wound healing is a highly dynamic process characterized by the coordinated interplay of various cell types, regulated meticulously by numerous wound-inducing mediators, THBS1 could regulate them in different processes. THBS1 expression is typically low in normal skin but is acutely upregulated following injury. This protein is produced by several cell types, including keratinocytes, fibroblasts, endothelial cells, and macrophages, and its expression returns to basal levels once inflammation subsides (82). In epidermal keratinocytes, those that secrete THBS1 differentiate following injury. These keratinocytes become polarized and migrate from the base to the wound surface, promoting epidermal wound healing. Notably, THBS1 gene knockdown delays this healing process (83). In neutrophils and macrophages, injury receptors expressing calcitonin gene-related peptide (CGRP) proliferate into granulation tissue post-injury, inducing THBS1 release. The deposition of THBS1 in the injured tissue environment inhibits the migration of neutrophils and macrophages while accelerating their cell death response to inflammatory factors. Simultaneously, THBS1 promotes macrophage secretion of cytokines and soluble growth factors, activates latent TGF-*β*, and inhibits fibrinolytic pathway proteolytic enzymes (including plasmin and urokinase-type plasminogen activators), thereby facilitating tissue repair (14, 84). Additionally, the upregulation of THBS1 enhances the differentiation of skin-derived precursors (SKPs) into fibroblasts, further promoting skin regeneration after injury (85). However, Experimental evidence from murine models with targeted dermal THBS1 overexpression demonstrates impaired healing kinetics during full-thickness cutaneous wound closure, mediated through interference with fibroblast migratory capacity and compromised neoangiogenesis within the wound bed (86). In ischemic tissue and transplanted flaps, THBS1 interacts with CD47 to downregulate the expression of VEGF and VEGFR2, exhibiting strong anti-angiogenic properties. This interaction restricts the angiogenesis and vasodilation activity of vascular endothelial cells’ nitric oxide signaling, adversely affecting tissue perfusion recovery after ischemic injury and resulting in reduced skin blood flow. Consequently, this significantly delays wound healing and related angiogenesis, ultimately decreasing the survival rate of the flap (87–89).

## 5 THBS1 and fibrosis

Fibrosis is characterized by the excessive and disordered deposition of collagenous ECM by myofibroblasts in response to tissue damage and subsequent repair mechanisms. THBS1 influences several biological processes associated with fibrosis, such as enhanced collagen secretion, pathological ECM deposition, cell migration, cell proliferation, and resistance to apoptosis, thereby serving as a critical regulator of fibrotic diseases (90).

Empirical studies have demonstrated that THBS1 collaborates with TGF-*β* in mediating the fibrotic processes across various systemic organs, including conditions such as recessive dystrophic epidermolysis bullosa (RDEB), scar formation, and systemic sclerosis (SSc). In the early stages of fibrosis, THBS1 primarily acts as a “pro-inflammatory and fibrotic initiator,” exhibiting rapid and significant upregulation. It activates latent TGF-β1, thereby triggering core fibrotic signaling pathways (91). During later stages, THBS1 functions more as a “matrix-stabilizing and persistence regulator,” often maintaining elevated expression levels with relatively minor fluctuations. Early diagnosis and intervention are critical; future approaches may focus on biomarkers, such as circulating THBS1 levels, to guide clinical decisions. Beyond THBS1, additional pathways—including those mediated by MMPs and ROS—can also activate TGF-*β* and exacerbate fibrosis (92). Since targeting THBS1 alone may be counteracted by compensatory activation of alternative routes, combination therapies (e.g., with TGF-β receptor inhibitors) could offer superior efficacy (93, 94). Notably, THBS1 plays an essential role in physiological wound healing. Therefore, long-term systemic inhibition of THBS1 risks impairing normal tissue repair. Enhancing treatment safety represents a key direction for future research, which may involve developing localized delivery strategies (e.g., inhalation for pulmonary fibrosis, topical administration for skin scars) or conditionally activated agents.

Research on antifibrotic interventions targeting the THBS1 pathway has advanced from fundamental mechanistic exploration into the early stage of clinical translation. Three main therapeutic strategies are being pursued: direct targeting, indirect regulation, and mechanism-based combination intervention. Among these, the direct targeting approach holds the greatest translational promise. The peptide inhibitor HTPEP-001, which specifically blocks THBS1 binding to CD36, has completed a Phase I clinical trial in pulmonary fibrosis. Its core mechanism lies in inhibiting THBS1-mediated activation of the key profibrotic factor TGF-β1 at its source. At the level of indirect regulation, strategies primarily involve modulating upstream molecules—such as using microRNA let-7f-5p to target and degrade THBS1 mRNA—or interfering with downstream receptors, for example, by employing CD36-neutralizing antibodies, to indirectly attenuate the function of the THBS1 pathway (95). The mechanism-based combination strategy utilizes drugs such as naltrexone to activate antioxidant pathways, thereby indirectly reducing THBS1 expression (96).

### 5.1 Recessive dystrophic epidermolysis bullosa

Recessive dystrophic epidermolysis bullosa is typified by pronounced skin fragility, chronic non-healing wounds, and heightened scarring and fibrosis. Within human fibroblasts, type VII collagen (C7) interacts with THBS1. In individuals with RDEB, mutations in the gene encoding C7 result in elevated levels of unbound THBS1 within fibroblasts. This increase facilitates the binding of THBS1 to LAP, thereby promoting the activation of latent TGF-*β* within the microenvironment (97). Activated TGF-β induces mesenchymal cells to transdifferentiate into myofibroblasts and upregulates the expression of fibrotic markers, including collagen, via the TGF-β/SMAD signaling pathway, while also augmenting excessive synthesis and abnormal accumulation of ECM proteins. The elevated levels of ECM proteins subsequently alter the bioavailability of TGF-β, thereby perpetuating a self-reinforcing cycle of TGF-β signaling within the skin, which contributes to fibrosis. Notably, the knockout of THBS1 results in a significant reduction of phosphorylated SMAD3 (p-SMAD3) in fibroblasts (98).

### 5.2 Scars

A keloid is a benign fibroproliferative disorder characterized by the abnormal proliferation of masses extending beyond the boundaries of the original skin lesions. The pathogenesis of keloids involves increased fibroblast proliferation and excessive deposition of type I collagen within the scar tissue. THBS1 plays a crucial role in the healing of skin tissue injuries and is closely associated with post-injury scar formation. Notably, the expression of THBS1 is significantly elevated in fibroblasts within scar tissue compared to normal skin. THBS1 knockout mice exhibit reduced skin collagen cross-linking. Mechanically, THBS1 facilitates fibroblast proliferation and extracellular matrix deposition by upregulating the IL6/JAK2/STAT3 pathway (12). Additionally, elevated levels of THBS1 mediate the activation of latent TGF-*β*, a potent inducer of type I collagen synthesis. TGF-β not only stimulates the recruitment of immune cells to the wound site and promotes extracellular matrix production but also induces fibroblast differentiation. MiR-205 downregulates the expression and activity of THBS1 and inhibits the growth and migration of fibroblasts. Additionally, the LSKL peptide has been shown to inhibit the overexpression and contractility of the fibroblast extracellular matrix, thereby impeding the development of hypertrophic scars (HTS). Consequently, miR-205 and the LSKL peptide present a promising novel strategy for the prevention or treatment of HTS (99, 100).

### 5.3 Systemic sclerosis

Systemic sclerosis is a chronic autoimmune disease characterized by extensive fibrosis of the skin and multiple internal organs, along with microvascular damage. The pathogenesis of SSc vascular disease involves an imbalance between angiogenic factors and vascular inhibitory factors, with THBS1 acting as a vascular inhibitory factor. SSc fibroblasts exhibit upregulated expression of the transcriptome marker THBS1 (101), which is considered to be a key gene related to skin fibrosis in the PI3K-AKT pathway, and inhibition of THBS1 can effectively reverse or alleviate organ fibrosis *in vivo* (102). The clinical progression of SCC is closely related to the increase in collagen production and the appearance and activation of dermal myofibroblasts. TGF-*β* is the factor most closely linked to the development of myofibroblasts, and THBS1 is a known upstream activator of TGF-β. Following treatment with anti-TGF-β/fresolimumab in SSc patients, the expression of THBS1 in the skin of these patients was significantly downregulated (103).

## 6 THBS1 and skin inflammation

THBS1 is a crucial endogenous regulator of inflammation. Initially, it was believed that increased THBS1 levels activate the TGF-β/SMAD pathway, enhancing the permeability of human dermal microvascular endothelial cells (HDMECs) to mast cells, and form a vicious cycle of “permeability enhancement—mast cell activation—medium release,” which further aggravates the inflammatory response (104). However, recent findings indicate that elevated THBS1 may also contribute to resistance against inflammation, with its deficiency leading to impaired wound healing and prolonged inflammation. During inflammatory development, THBS1 induces a chemotactic response to injured tissue, inhibits the migration of neutrophils and macrophages, promotes the phagocytosis of injured and necrotic cells, suppresses Th17 differentiation, and encourages the transformation of macrophages into the anti-inflammatory and pro-repair M2 phenotype (14). In this regard, we propose that the role of THBS1 differs between acute and chronic skin inflammation, with its function transitioning from a “coordinated repair mediator” in the acute phase to an “inflammation sustainer” in the chronic phase. The core of this shift lies in the specific regulation of different immune cell subsets by THBS1. During acute inflammation, THBS1 primarily drives the resolution phase by promoting apoptosis and clearance of early infiltrating cells, such as neutrophils, and by polarizing macrophages from a pro-inflammatory (M1) toward a repair-associated (M2) phenotype, thereby initiating tissue repair and coordinating timely inflammation resolution (105). However, in chronic inflammatory settings—such as psoriasis and atopic dermatitis—persistently elevated inflammatory cytokines (e.g., IL-17, TNF-*α*, and IL-23) create a hypoxic and highly oxidative stress milieu, which further induces THBS1 expression. At this stage, THBS1 acts through receptors such as CD47 to continuously modulate resident immune cells: it suppresses full activation and antigen presentation by dendritic cells; impairs the differentiation and function of regulatory T cells (Tregs); and promotes macrophages to adopt a pro-fibrotic M2-like state, shifting from physiological repair mediators to pathological disruptors (106). These specific inhibitory and modulatory effects on both innate and adaptive immune cells collectively establish a microenvironment that sustains immune activation, drives pathological remodeling, and facilitates abnormal angiogenesis, thereby promoting chronicity of inflammation. The immune microenvironment plays a critical role in regulating this dual functionality of THBS1. THBS1 expression is increased in the skin of patients with contact dermatitis, where it downregulates delayed-type skin hypersensitivity and reduces edema formation, vascular permeability, vascular remodeling, and neutrophil infiltration associated with contact hypersensitivity (13, 107). However, reduced THBS1 and CD47 mRNA expression was observed in the skin of psoriasis patients, with THBS1 expression showing a negative correlation with both the extent of psoriatic lesions and disease activity (108). Reduced THBS1 expression has been shown to inhibit the differentiation of CD4 + T cells into Treg cells and to hinder latent TGF-*β* activation (109). TGF-β functions as a growth inhibitor for keratinocytes; decreased regulation of its growth activity can result in abnormal cell proliferation in patients with psoriasis (110, 111). We hypothesize that the expression of THBS1 in psoriasis is not simply characterized as “elevated” or “reduced” but rather exhibits dynamic regulation. In this context, the prolonged chronic inflammatory environment establishes a pathological steady state with relatively low THBS1 expression, which does not represent the activation of a pro-repair signal. In chronic inflammatory diseases of genital skin, such as lichen sclerosus (LS), researchers have identified THBS1 hypermethylation, which is consistent with findings in vulvar squamous cell carcinoma (SCC) (112). In the context of infectious immune diseases, including bacterial infections, THBS1 has been demonstrated to impede the development of antimicrobial resistance in skin commensal bacteria and markedly decrease proinflammatory cytokines such as IL-1β, IL-6, IL-8, and TNF-*α*, thereby exhibiting potential to mitigate the inflammatory response to bacterial infection (113). Notably, a recent *in vivo* study on inflammatory pain conducted by Jain et al. revealed that immune cells in three distinct pain models were responsible for the production and secretion of THBS1, indicating that THBS1 is not only associated with inflammation but also plays a role in pain regulation, showing the potential to inhibit pain signals through interaction with nociceptors (114).

## 7 THBS1 and skin tumors

Tumor secretory protein (PSAP) functions as a paracrine inhibitor of both primary and metastatic tumor growth, primarily by inducing the expression of THBS1 in myeloid-derived suppressor cells (MDSCs). Concurrently, the anti-angiogenic properties of THBS1 are also thought to play a significant role in inhibiting tumor growth. Sui et al. proposed enhancing THBS1 expression in the tumor microenvironment by blocking serine protease 2 (PRSS2)-mediated inhibition of THBS1 as a potential cancer treatment (115). However, the role of THBS1 in tumorigenesis is complex and contradictory. Numerous studies have demonstrated that THBS1 can inhibit angiogenesis and tumor growth in lung and bladder cancers, whereas its role in breast, pancreatic, and gastric cancers appears to be the opposite. Additionally, conflicting data have been reported regarding the role of THBS1 in the growth and metastasis of skin tumors.

Basal cell carcinoma (BCC), SCC, and malignant melanoma (MM) are among the most prevalent skin tumors. Researches have indicated that THBS1 is a potential tumor suppressor. THBS1 can inhibit tumor angiogenesis by inhibiting the pro-angiogenesis factor signaling pathway (NO, VEGF, etc.) and binding with CD36 to mediate the apoptosis of vascular endothelial cells. Skin squamous cell carcinoma Scl-1 cells deficient in a copy of chromosome 15 exhibit highly malignant behavior; meanwhile the same cells transfected with THBS1 demonstrate benign growth characteristics (116, 117). Overexpression of THBS1 significantly reduced intradermal tumor growth in A431 epidermoid carcinoma cells and SCC-13 squamous cell carcinoma (118). These findings suggest that THBS1 has the potential to entirely prevent the development of cutaneous squamous cell carcinoma. However, the role of THBS1 in tumors is dual-faceted and cannot be universally characterized. In squamous cell carcinoma 11B cell line, decreased THBS1 secretion inhibited tumor growth *in vivo*, whereas overexpression of THBS1 may result in enhanced tumor proliferation. This is also true in CTCL, where correlated with an increased risk of disease-related mortality. Compared to normal CD4 + T cells, CTCL tumor cells and CTCL cell lines (Hu78, HH, and MyLa cells) exhibit elevated expression of CD47. THBS1 interacts with CD47 to facilitate the migration, proliferation, and survival of CTCL tumor cells, and these effects can be mitigated by anti-CD47 neutralizing antibodies or CD47 knockdown (16). THBS1 expression is higher in primary melanoma and metastasis than in common and dysplastic nevus, and THBS1 determines the invasion and metastasis of melanoma (119). As a target gene of YAP, which has been identified as a key regulator of melanoma metastasis, THBS1 has been observed to exhibit promoter hypermethylation in cases of melanoma brain metastasis (48, 120). THBS1 is thus considered a critical mediator of melanoma cell invasion and is associated with poor prognosis. It has been identified as a biomarker with high specificity for highly invasive melanoma (121). Research indicates that malignant cells that exhibit typical epithelial-mesenchymal transformation (EMT) characteristics in mouse and human melanoma models partially establish an immunosuppressive microenvironment by secreting THBS1, drive clonal expansion of regulatory T cells (TREGs), and induce dendritic cells to acquire a functional defect phenotype. Administration of anti-THBS1 monoclonal antibodies via intratumoral injection has been shown to enhance tumor-specific infiltrating lymphocytes and systemic immune responses, thereby significantly inhibiting tumor growth and metastasis (122). However, several studies have indicated that intratumoral electrotransfer of anti-angiogenic THBS1 plasmids can significantly inhibit tumor growth. As an anti-angiogenic agent, THBS1 plasmid electrotransfer presents a promising therapeutic approach for melanoma treatment (123).

**Figure 4:**
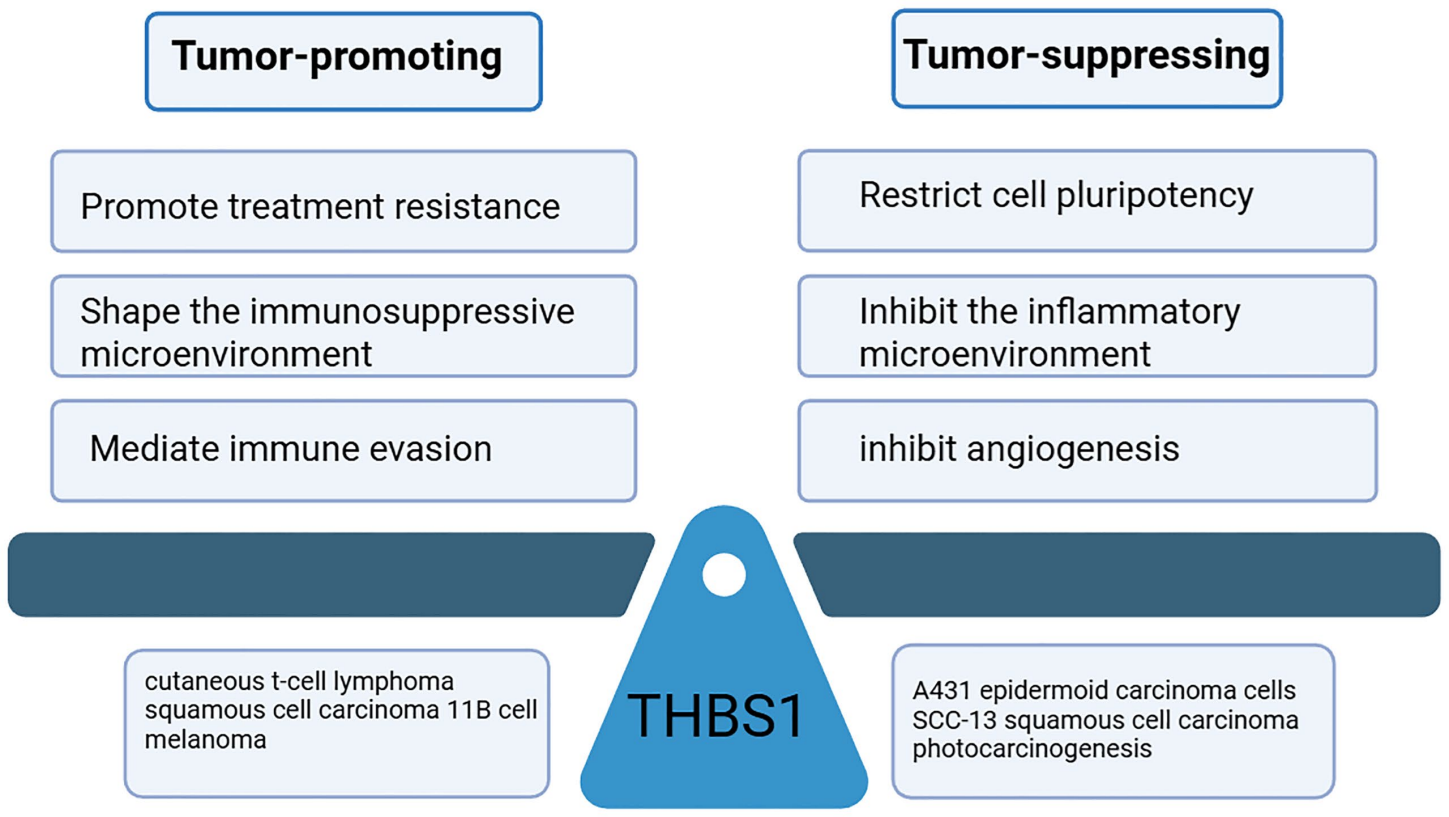
The role of THBS1 in skin tumors.

The ultimate biological effect of THBS1 is determined not only by its cellular origin but is also shaped collectively by its specific receptor engagement and TME (Figure 4). During the early stages of tumorigenesis or in a preventive context, THBS1 is primarily expressed by epithelial or endothelial cells, where it exerts tumor-suppressive functions by establishing a microenvironment unfavorable for tumor initiation and growth. Anti-angiogenic activity represents the most classical tumor-suppressive mechanism of THBS1. By binding with high affinity to receptors such as CD36 on endothelial cells, THBS1 activates signaling pathways that induce endothelial apoptosis and inhibit migration (1). This effectively blocks the formation of new blood vessels, thereby depriving the tumor of the oxygen and nutrients essential for its growth. Concurrently, THBS1 significantly suppresses the migration and activation of leukocytes into tissues and downregulates the production of key pro-inflammatory cytokines such as IL-6 and IL-12 (80). Through this anti-inflammatory action, THBS1 reduces the genomic instability and aberrant proliferative pressure induced by inflammatory mediators in the microenvironment, thereby contributing to cancer prevention. Furthermore, by interacting with cell-surface receptors such as CD47 and integrins, THBS1 indirectly inhibits the activity of core transcription factors like c-Myc, limiting the proliferative and regenerative capacity of tumor cells. However, when tumors progress beyond early constraints, tumor cells and tumor-associated stromal cells—such as cancer-associated fibroblasts and M2-polarized tumor-associated macrophages—secrete large amounts of THBS1, shifting its function toward promoting tumor survival and invasion. The core pro-tumorigenic mechanism of THBS1 lies in mediating immune evasion. By binding to the receptor CD47 on immune cells (124), particularly macrophages and T cells, THBS1 blocks the phagocytic clearance of tumor cells by macrophages. Moreover, sustained activation of the THBS1-CD47 axis can lead to functional exhaustion of tumor-infiltrating T cells, further dismantling adaptive immune attacks and enabling tumors to survive and expand under immune surveillance (125). In conclusion, the study of the function of THBS1 requires a dynamic analysis in the context of the environment. The “either non-oncogene or oncogene” binary view must be abandoned.

## 8 Summary

Since the discovery of THBS1 over three decades ago, significant advancements have been made in elucidating its structure and functional mechanisms. THBS1 plays a crucial role in regulating the synthesis and degradation of the extracellular matrix, facilitating cell migration and proliferation, and interacting with other proteins to modulate immune and inflammatory responses in the skin. THBS1 significantly influences the structural and functional integrity of the skin and holds considerable promise in the field of dermatology. However, as research progresses, the dualistic and often contradictory nature of THBS1 function—particularly in the contexts of cutaneous inflammation and tumorigenesis—has become increasingly apparent. Although numerous studies have examined the dual roles of thrombospondin-1 (THBS1) in tumor angiogenesis and immunomodulation, the research landscape remains largely uncharted when focusing specifically on the skin as a distinct organ. A primary knowledge gap lies in the precise, cell-type-specific actions of THBS1 within the cutaneous microenvironment. While it is known that THBS1 can be secreted by keratinocytes, fibroblasts, immune cells, and others, the exact correspondence between its cellular origin and its function in different contexts—such as skin homeostasis, aging, carcinogenesis, and wound healing—has not been fully elucidated. Secondly, the role of THBS1 in non-neoplastic cutaneous pathologies has emerged as a growing focus, especially its connection to epigenetic memory and tissue repair. A breakthrough study in 2024 revealed that skin fibroblasts previously exposed to radiotherapy retain an “epigenetic memory,” characterized by persistently increased chromatin accessibility at the THBS1 gene locus (126). Upon subsequent injury, these cells overexpress THBS1, which in turn suppresses fibroblast migration and contractility, thereby impairing wound healing; conversely, anti-THBS1 antibody treatment promoted repair. This raises a critical question: whether THBS1-associated epigenetic reprogramming also occurs in photoaging and chronic inflammatory skin diseases, and how it may link to wound healing and the initiation or progression of cutaneous tumors. Exploring THBS1 as a potential “molecular bridge” connecting past insults (e.g., UV exposure, radiation) to current skin dysfunction represents an important future direction. Finally, at the translational medicine level, targeting THBS1 and its pathways for therapeutic intervention remains a major challenge. The dual nature and the context-dependent nature of THBS1’s function make simple “activation” or “inhibition” strategies likely to lead to unpredictable consequences. Future research directions should focus on developing precise regulation, analyzing the specific microenvironmental signals that drive the functional transformation of THBS1, and designing targeted blocking of THBS1 binding to receptors to locally regulate activity, to achieve breakthrough progress in the treatment of clinical skin diseases.
